# Close correlation between the ankle-brachial index and symptoms of depression in hemodialysis patients

**DOI:** 10.1007/s11255-017-1598-7

**Published:** 2017-04-28

**Authors:** Ing-Chin Jong, Hung-Bin Tsai, Chien-Hung Lin, Tsung-Liang Ma, How-Ran Guo, Peir-Haur Hung, Kuan-Yu Hung

**Affiliations:** 1 Division of Nephrology, Department of Internal Medicine, Ditmanson Medical Foundation Chia-Yi Christian Hospital, 539 Jhongsiao Road, Chia-Yi City, 600 Taiwan; 2 Department of Tramatology, National Taiwan University Hospital, 7 Chung Shan S. Road, Zhongzheng Dist., Taipei City, 10002 Taiwan; 3 Institute of Clinical Medicine, National Yang-Ming University, 155 Linong Street, Taipei City, 112 Taiwan; 4 Department of Pediatrics, Zhongxing Branch, Taipei City Hospital, 145 Zhengzhou Road, Datong Dist., Taipei City, 10341 Taiwan; 5 Department of Environmental and Occupational Health Medical Colleage, National Cheng Kung University, No. 1 University Road, Tainan City, 70401 Taiwan; 6 Department of Applied Life Science and Health, Chia-Nan University of Pharmacy and Science, 60 Erren Road, Rende Dist., Tainan City, 71710 Taiwan; 7 Department of Internal Medicinem, National Taiwan University Hospital, Hsin-Chu Branch, 25 Jingguo Road, Hsin-Chu City, 300 Taiwan

**Keywords:** Ankle-brachial index, Beck depression inventory, Depression, Hemodialysis

## Abstract

**Background:**

As both of peripheral arterial disease (PAD) and depression carried a poor prognosis in patients on maintenance hemodialysis (MHD), we investigated the correlation between the ankle-brachial index (ABI), an indicator of subclinical PAD, and symptoms of depression in patients on MHD.

**Methods:**

One hundred and twenty-nine patients on MHD (75 males and 54 females, mean age 64.8 ± 12 years) were enrolled in this cross-sectional study, which aimed at evaluating the relationship between symptoms of depression and ABI. Demographic as well as clinical and laboratory variables including status of diabetes, chronic hepatitis C infection, dialysis duration, Charlson comorbidity index (CCI), plasma levels of albumin, C-peptide, insulin, high-sensitive C-reactive protein (hsCRP), interleukin-6 (IL-6), adiponectin, and lipid profile were obtained. The self-administered beck depression inventory (BDI) was used to determine the presence or absence of symptoms of depression, and depression was defined as a BDI score ≧14. Multivariable-adjusted linear regression models were constructed to confirm the independent association of biologic parameters of symptoms of depression. Significance was defined as *P* < 0.05. Statistical analyses were performed using SPSS/Windows software (SPSS Science, v. 15.0, Chicago, IL).

**Results:**

The mode of multivariate analysis showed that diabetes (*β* = 3.594; *P* = 0.040), hepatitis C infection (*β* = 4.057; *P* = 0.008), levels of serum albumin (*β* = −5.656; *P* = 0.024), C-peptide (*β* = −0.292; *P* = 0.002), ABI (*β* = −9.041; *P* = 0.031), and Ln-transformed hsCRP were significantly associated with BDI.

**Conclusions:**

Hepatitis C infection, serum levels of albumin, C-peptide, and ABI levels were found to be correlated with BDI (*P* < 0.05).

## Introduction

Depression is highly prevalent throughout the world and is increasing in the range of 8–12% per year [1]. Among patients with chronic medical illness, the annual prevalence rate of depression is significantly higher, approximately 25% per year [2]. It is generally accepted by the practitioners that depression is the most common psychological problem in patients with end stage renal disease (ESRD) and patient on maintenance hemodialysis (MHD) with depression carries a poor prognosis [3]. Depression is often considered the final common pathway for different disease processes that occur across the biopsychosocial continuum. In patients with ESRD, contributing factors to the onset of depression include physical and emotional stress related to functional limitations, dietary constraints, time restrictions to dialysis, medical comorbidities, and adverse effects of medication [4–6].

MIA syndrome (malnutrition, inflammation, and atherosclerosis) may also contribute to the symptoms of depression in patients with ESRD. Recent available data suggest that peripheral arterial disease (PAD) is prevalent in hemodialysis (HD) patients and is a strong predictor for subsequent cardiovascular disease and overall mortality [7]. The ankle-brachial index (ABI) has been reported as a good marker for atherosclerosis and useful for the diagnosis of PAD [8]. Although traditional cardiovascular risk factors such as smoking, diabetes, hypertension, and hyperlipidemia have been proven to be strong risk factors for PAD in the general population [9], depression is associated with PAD in women, and is not unusual in the general population having suffered from PAD, and occurs in patients with diabetes [10–13].

Previous studies have shown that risk factors for depressive symptoms in patients on MHD may include poor sleep quality, pruritus, hypoalbuminemia, higher serum levels of IL-6, C-reactive protein, and comorbidities [14, 15]. However, the impact of PAD in the development of symptoms of depression in patients on MHD has not been evaluated. We currently hypothesize that ABI is correlated with the symptoms of depression in patients on MHD with the purpose of early detection of the risk factors of the depressive disorder in patients on MHD. Since depression is correlated with increased mortality rate in patients on MHD [3], identification of factors correlated with the symptoms of depression may allow clinicians to further optimize their treatment strategies.

## Materials and methods

### Study population

Two hundred and four patients with ESRD in a regional hospital St. Martin De Porres Hospital (Chiayi City, Taiwan) were recruited for this cross-sectional study.

### Inclusion and exclusion criteria

#### Inclusion criteria

Those who had received HD treatment 3 times per week for greater than 3 months, with each session lasting for 4 h, were included in this study. Informed consent was secured for all study subjects. This clinical study followed the Declaration of Helsinki and was approved by the Ethics Committee of St. Martin De Porres Hospital.

#### Exclusion criteria

Patients with irregular or inadequate HD therapy with a mean Kt/V < 1.2, or with a HD duration <3 months before entry, or patients found to have major infections, amputation of lower extremity, an ABI ≧ 1.3, or patients who had been taking immunosuppressive agents for at least 1 month were excluded. Patients with incomplete studies were also excluded.

### Study evaluations

Demographic data such as age and sex were obtained using a questionnaire. Clinical characteristics, including medical comorbidities, current medications, and recent laboratory examinations were obtained using patient medical records. Charlson comorbidity index (CCI) was used to evaluate comorbid diseases severity in enrolled patients [16]. The beck depression inventory (BDI) was distributed by a healthcare assistant during a HD session, and the score for BDI was self-administered and self-reported. The presence and/or severity of depression was categorized by BDI score for the general population without ESRD: depression symptoms (14–63) and non-depression symptoms (0–13), owing to the absence of standard BDI categories for patients with ESRD [17].

Hematological and biochemical parameters were evaluated using blood obtained from midweek pre-dialysis samples. Plasma samples were separated from blood cells and stored at −70 °C. Venous blood samples were collected the morning after an overnight fast. Samples were centrifuged at 1500×*g* at 4 °C for 10 min for analysis. Kt/V was calculated using Daugirdas’ second formula [18].

Hematologic levels were measured using Sysmex XT-1800i hematology analyzer (Sysmex America Inc., Mundelein, IL). Levels of serum high-sensitivity C-reactive protein (hsCRP) and insulin were measured using a chemiluminescent immunoassay (Immulite 2000; DPC, Los Angeles, CA). Insulin sensitivity was quantified using the homeostasis model assessment of insulin resistance (HOMA-IR) equation to measure fasting insulin and glucose levels (HOMA-IR = I × 3 G/22.5), where ‘I’ is insulin (IU/mL) and ‘G’ is glucose (mmol/L) (IR: HOMA-index ≧ 2.5μU/mL × mmol/L) [19]. Levels of serum C-peptide were measured using two-site sandwich immunoassay automated chemiluminescence system (Bayer ADVIA Centaur XP). Fasting blood sugar, calcium, phosphate, albumin, glutamic pyruvic transaminase (GPT), cholesterol, and triglyceride levels were measured using an automated analyzer (Hitachi 7170, Tokyo, Japan). Serum intact parathyroid hormone **(**iPTH) levels were measured using immunoradiometric assays (Nichols Institute Diagnostics, San Juan Capistrano, CA, USA). For hsCRP, the intra-assay coefficient of variance was 8.7%, sensitivity was 0.1 mg/L, and upper limit of detection was 150 mg/L [20]. Expected values for healthy individuals were hsCRP ≦ 3 mg/L [21]. Serum proinflammatory cytokine levels were measured using a high-sensitivity interleukin (IL)-6, tumor necrosis factor (TNF)-α, and adiponectin immunoassay kits. These measurements were based on a solid-phase sandwich enzyme-linked immunoassay with TNF-α (normal range 0.5–100 pg/mL; RayBiotech), recombinant human IL-6 (normal range 0.03–200 pg/mL; RayBiotech, Atlanta, GA), and adiponectin (normal range 0.5–100 pg/mL; RayBiotech). Anti-hepatitis C virus (Anti-HCV) antibodies were measured using a third-generation enzyme immunoassay (Abbott Laboratories, North Chicago, IL).

The ABI index and pulse wave velocity (PWV) were measured in all patients by applying a vascular screening device (VP 1000; Colin Corp Co, Ltd, Komaki, Japan) that simultaneously measures bilateral arm and ankle (brachial and posterior tibial arteries, respectively) blood pressure. Measurements of both the ABI index and PWV were obtained after completion of the dialysis treatment and after allowing patients to rest in a supine position for at least 5 min. Some patients required more than 10 min for their blood pressure to stabilize. ABI was calculated using the ratio of ankle systolic pressure to arm systolic pressure. Both the systolic pressure of the arm without dialysis access and the lower value of the ankle pressure were used to calculate the ABI index. The ABI index for each patient was checked at least twice during each dialysis session, with the mean used for subsequent analysis. A diagnosis of PAD required an ABI < 0.9, which may be indicative of varying degrees of atherosclerosis in the arteries of the lower extremity. Patients with an ABI ≧ 1.3 were excluded, because this implies poorly compressible leg arteries and inability to accurately gauge arterial obstruction [7]. For measuring PWV, pulse waves obtained from the brachial and tibial arteries were recorded simultaneously, and the transmission time, defined as the time interval between the initial increase in brachial and tibial waveforms, was determined. The transmission distance from the arm to each ankle was calculated according to body height. The PWV value was automatically computed as the transmission distance divided by the transmission time. After obtaining bilateral PWV values, the highest one was used as representative for each subject. The PWV measurement was done once in each patient.

### Statistical analysis

Continuous variables were expressed as mean ± SD, and categorical variables were expressed as percentages. Data were analyzed using a *t* test or *χ* ^2^ test, depending on the nature of the variables. Proinflammatory cytokine concentration was Ln-transformed to improve its level of normality. A Spearman’s correlation analysis was performed to evaluate the relationship between biological markers and symptoms of depression. Multivariable-adjusted linear regression models were constructed to confirm the independent association of biologic parameters of symptoms of depression. Significance was defined as *P* < 0.05. Statistical analyses were performed using SPSS/Windows software (SPSS Science, v. 15.0, Chicago, IL).

## Results

**Fig. 1:**
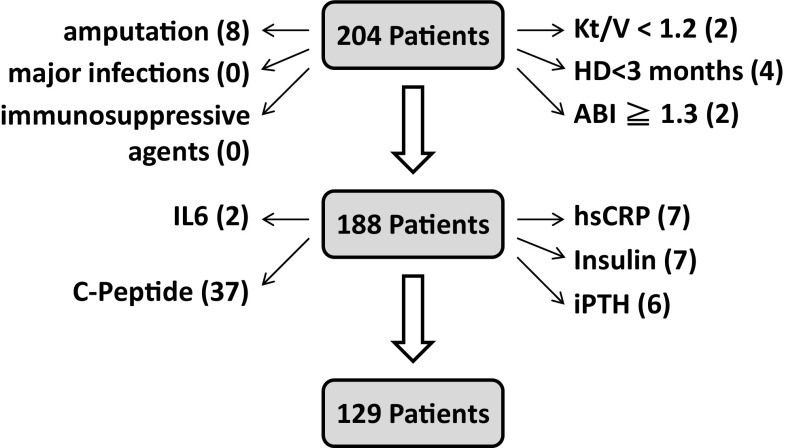
Study flow diagram

**Fig. 2:**
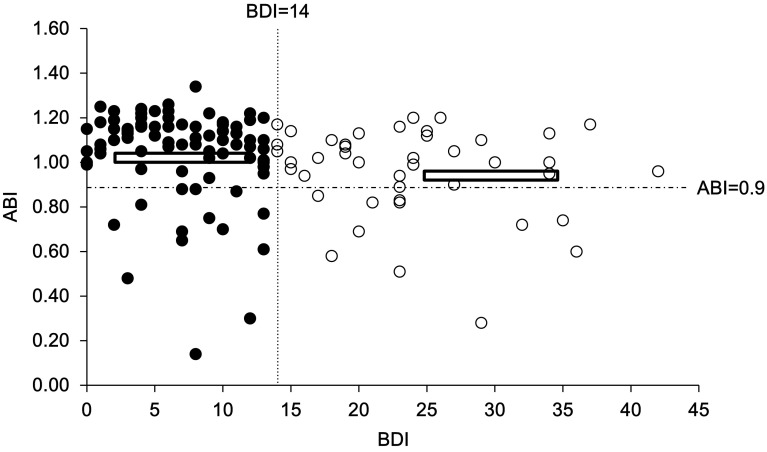
Scatterplots diagram demonstrated the distribution between the status of ABI and the BDI scores. The difference of the status of ABI between patients with BDI scores ≧ 14 and those with BDI scores < 14 is statistically significant (*P* < 0.05)

As shown in the study flow diagram Fig. 1, a total of 204 patients were recruited, of which four patients with hemodialysis duration less than 3 months, two patients with Kt/V less than 1.2, two patients with ABI ≧ 1.3, eight patients with amputation of lower extremity, 59 patients with incomplete studies including 37 patients without data of serum C-peptide levels, 6 patients without data of serum iPTH levels, 7 patients without data of serum insulin levels, 7 patients without data of serum hsCRP levels, and 2 patients without data of serum IL-6 levels were excluded. The resulting 129 patients were enrolled in the study. Among enrolled patients, 33.3% were depressed and 66.7% were not depressed (non-depressed), respectively (Table 1). Clinical and biochemical characteristics in depressed and non-depressed HD patients are summarized in Table 1. HD patients in depressed group were significantly older than the non-depressed group (66.7 ± 10.1 vs. 60.8 ± 13.9; *P* = 0.007). The depressed group had a significantly higher prevalence of hepatitis C (58.1 vs. 39.5%; *P* = 0.046) and diabetes (74.4 vs. 39.5%; *P* < 0.001) compared with the non-depressed group. The biochemical examination showed that depressed patients had significantly lower levels of albumin (3.7 ± 0.3 vs. 4.0 ± 0.4; *P* < 0.001), C-peptide (10.6 ± 6.5 vs. 13.6 ± 10.3; *P* = 0.044), and ABI (0.96 ± 0.2 vs. 1.04 ± 0.2; *P* = 0.028) compared with the non-depressed group. The distribution of the status of ABI and BDI scores was demonstrated in Fig. 2. Higher CCI scores (4.0 ± 2.3) found in the depressed group than that found in the non-depressed group CCI scores (2.5 ± 2.2) are indicative of the association of depression with CCI scores in patients on MHD. Levels of depression and the other related variables in HD patients are reported in Table 2. The BDI was found to be positively correlated with age (*r* = 0.348; *P* < 0.001) and CCI (*r* = 0.387; *P* < 0.001). In addition, BDI was significantly negatively correlated with levels of serum albumin (*r* = −0.399; *P* < 0.001) and ABI (*r* = −0.325; *P* < 0.001). The mode of multivariate analysis showed that diabetes (*β* = 3.594; *P* = 0.040), hepatitis C infection (*β* = 4.057; *P* = 0.008), levels of serum albumin (*β* = −5.656; *P* = 0.024), C-peptide (*β* = −0.292; *P* = 0.002), ABI (*β* = −9.041; *P* = 0.031), and Ln-transformed hsCRP were significantly associated with BDI as shown in Table 3. However, gender and age were not found to be associated with BDI.

**Table 1:** Differences of clinical and biochemical parameters between depressed and non-depressed hemodialysis patients

	BDI < 14 (*n* = 86)	BDI ≧ 14 (*n* = 43)	*P* value
Age (years)	60.8 ± 13.9	66.7 ± 10.1	0.007*
Male (%)	50 (58.1%)	25 (58.1%)	1.000
Dialysis duration (years)	4.8 ± 3.9	5.0 ± 4.3	0.721
Body mass index (kg/m^2^)	23.1 ± 4.0	22.9 ± 3.1	0.775
Systolic blood pressure (mmHg)	139.9 ± 19.6	136.3 ± 14.8	0.289
Diastolic blood pressure (mmHg)	75.9 ± 8.4	75.4 ± 6.0	0.719
Hepatitis C infection (%)	34 (39.5%)	25 (58.1%)	0.046*
Diabetes (%)	34 (39.5%)	32 (74.4%)	<0.001*
Albumin (g/dl)	4.0 ± 0.4	3.7 ± 0.3	<0.001*
Hemoglobulin (gm/dl)	10.4 ± 1.5	10.4 ± 1.7	1.000
ALT (U/L)	18.8 ± 12.8	29.4 ± 56.3	0.231
Fasting blood glucose (mg/dl)	113.5 ± 63.2	131.8 ± 71.6	0.141
Insulin (μIU/ml)	23.5 ± 33.3	25.8 ± 37.2	0.726
C-peptide (ng/mL)	13.6 ± 10.3	10.6 ± 6.5	0.044*
HOMA-IR (μU/mL × mmol/L)	6.2 ± 7.3	8.6 ± 10.9	0.140
iPTH (pg/mL)	187.2 ± 171.5	165.1 ± 169.1	0.490
Ca × P (mg^2^/dL^2^)	42.6 ± 13.9	42.2 ± 14.3	0.871
Total cholesterol (mg/dl)	170.9 ± 38.3	157.9 ± 36.7	0.067
Triglyceride (mg/dl)	167.4 ± 119.2	153.2 ± 123.1	0.528
HDL cholesterol (mg/dl)	44.5 ± 13.6	44.2 ± 16.3	0.911
LDL cholesterol (mg/dl)	97.8 ± 27.2	91.4 ± 23.7	0.196
Ln_hsCRP (mg/dL)	1.33 ± 1.28	1.34 ± 1.30	0.991
Ln_Adiponectin (pg/mL)	5.6 ± 0.3	5.7 ± 0.3	0.063
Ln_IL-6 (pg/mL)	2.8 ± 1.0	3.0 ± 0.8	0.488
Ln_TNF-α (pg/mL)	0.84 ± 0.96	0.82 ± 0.96	0.941
CCI	2.5 ± 2.2	4.0 ± 2.3	<0.001*
ABI	1.04 ± 0.20	0.96 ± 0.20	0.028*
PWV (m/s)	17.9 ± 5.7	18.4 ± 5.1	0.661

*ABI* ankle-brachial index, *BDI* beck depression inventory, *CCI* Charlson comorbidity index; *hsCRP* high-sensitivity C-reactive protein

* *P* < 0.05

**Table 2:** Spearman rank correlation between BDI levels and the other variables in hemodialysis patients (*n* = 129)

	*r*	*P* value
Age	0.348	<0.001*
Body mass index (kg/m^2^)	−0.044	0.619
CCI	0.387	<0.001*
*Blood pressure*		
Systolic	−0.078	0.380
Diastolic	−0.064	0.474
Albumin (g/dl)	−0.399	<0.001*
Glucose	0.090	0.311
*Plasma lipids*		
LDL	−0.115	0.193
HDL	−0.050	0.575
Triglycerides	−0.121	0.172
Insulin (μIU/ml)	0.044	0.626
C-peptide (ng/mL)	−0.175	0.047*
HOMA-IR (μU/mL × mmol/L)	0.076	0.391
ABI	−0.325	<0.001*
PWV (m/s)	0.098	0.271
Ln_hsCRP (mg/dL)	0.015	0.869
Ln_TNF-α (pg/mL)	−0.063	0.480
Ln_IL-6 (pg/mL)	0.143	0.105
Ln_Adiponectin (pg/mL)	0.160	0.069

*ABI* ankle-brachial index; *BDI* beck depression inventory, *CCI* Charlson comorbidity index; *hsCRP* high-sensitivity C-reactive protein, *PWV* pulse wave velocity

* *P* < 0.05

**Table 3:** Multivariable-adjusted linear regression analysis for the BDI score (*n* = 129)

Variables	Multivariate	
	Coefficient (*β*)	*P* value
Male gender	−0.849	0.593
Age (years)	0.011	0.874
Diabetes	3.594	0.040*
Hepatitis C infection	4.057	0.008*
Albumin (g/dl)	−5.656	0.024*
C-peptide	−0.292	0.002*
ABI	−9.041	0.031*
HOMA-IR	−0.015	0.914
Ln_hsCRP	−1.391	0.044*
Insulin	0.032	0.331
Ln_IL-6	1.126	0.231
Ln_TNF-α	−0.843	0.306

*ABI* ankle-brachial index, *BDI* beck depression inventory

* *P* < 0.05

## Discussion

In the current analysis of BDI scores, serum levels of albumin, C-peptide, chronic hepatitis C infection, diabetes, and ABI were all demonstrated to be correlated with symptoms of depression in patients on MHD. To the best of our knowledge, these results provide the first evidence for an association of ABI with symptoms of depression in patients on MHD.

Regardless of the statistical analysis used to obtain BDI score, patients with a BDI ≧ 14 had significantly lower serum albumin levels compared with patients with a BDI < 14. Previous studies have revealed a correlation between serum albumin levels with symptoms of depression in patients on MHD [14, 15]. Malnutrition in dialysis patients was not caused by poor intake alone, but also was the result of chronic inflammation [22]. In an animal model, it has been suggested that nuclear factor-kappa B (NF-kB) caused decreased albumin gene expression in states of inflammation, leading to a decreased rate of albumin synthesis and a reduced serum albumin concentration [23]. And in human study, it showed decreased liver synthesis of albumin in inflammation [24]. In the general population or for patients on MHD, infection with chronic hepatitis C has been demonstrated to be correlated with symptoms of depression [25, 26], consistent with the current findings.

It has been previously demonstrated that diabetic patients have a double risk of depression as compared to the nondiabetic group [27]. Furthermore, in patients on MHD, the presence of diabetes is significantly associated with symptoms of depression [14]. The current study also demonstrated that diabetes was correlated with the symptoms of depression in patients on MHD after the mode of the multivariable-adjusted linear regression analysis for the BDI score. C-peptide and insulin are secreted in equal amounts, and clinically monitoring of serum levels of C-peptide is considered to be a valid measure of insulin secretion in diabetic patients [28]. We suspect that diabetic patients with lower C-peptide serum levels and lower levels of endogenous insulin secretion might suffer from much more beta-cell failure due to the longer duration of illness associated with diabetes. Consistent with this previous report, we currently demonstrate a correlation between lower levels of serum C-peptide with symptoms of depression in patients on MHD.

A possible link between ABI and symptoms of depression in patients on MHD has been established. Smoking, diabetes, hypoalbuminemia, and dialysis duration have been demonstrated to be risk factors for PAD in patients on MHD [29, 30]. We also reported that diabetes, hypoalbuminemia, and serum levels of C-peptide were correlated with symptoms of depression in patients on MHD; however, they were also risk factors for PAD in patients on MHD. PAD and depression might be closely correlated, intimately interacting physiologically and psychologically. Also, in another point of view, the probable molecular link between ABI and BDI score might be through the proinflammatory cytokines IL-6 and CRP, influencing both the vasculature and brain. The reasons were as follows. Firstly, a prospective population-based cohort study by Tzoulaki et al. [31] demonstrated that the serum level of C-reactive protein (CRP) was not only correlated with PAD severity at baseline but also a predictor of progression of PAD in the general population. And our previous study also showed that IL-6 (a precursor of CRP) was associated with PAD in patients on MHD [32]. Secondly, in a prospective, population-based cohort study by Khandaker et al. [33], they demonstrated that children with higher serum levels of IL-6 and CRP would be exposed to increased future risks of depression and psychosis in young adulthood in a dose-dependent way. And previous study by Hung et al. [15] also revealed that serum levels of IL-6 and hsCRP were associated with symptoms of depression in patients on MHD. So, ABI and BDI scores might be closely correlated. MIA syndrome could be associated with the symptoms of depression in patients on MHD. Taken together, we demonstrated a correlation between ABI and the symptoms of depression in patients on MHD.

Limitations of the current study include the lack of inferences on causality due to the cross-sectional nature of the study; therefore, implications on possible mechanisms should be regarded as hypotheses. Secondly, the study was monocentric with a small number of enrolled subjects; however, we would like to emphasize that all subjects were diagnosed from a large HD population screened for symptoms of depression and PAD and the symptoms of PAD were asymptomatic and the presence of an initial disorder of the arteries in the lower limbs was unknown. Thirdly, the presence and/or severity of depression was categorized by using the BDI score, instead of a clinician-administered structured diagnostic interview, and there might be some misclassifications.

## Conclusions

In conclusion, to our knowledge, this is the first study providing evidence that ABI is closely correlated with symptoms of depression in patients on MHD. Furthermore, patients on MHD with symptoms of depression may have lower serum C-peptide, lower serum albumin levels, and lower ABI levels. Factors including diabetes, hepatitis C infection, and ABI levels may predispose the development of symptoms of depression. Further study is required to evaluate the association between depression and other health-related outcomes, including PAD.
